# Feasibility and utility of in-home body weight support harness system use in young children treated for spinal muscular atrophy: A single-arm prospective cohort study

**DOI:** 10.1371/journal.pone.0300244

**Published:** 2024-03-19

**Authors:** Megan A. Iammarino, Lindsay N. Alfano, Natalie F. Reash, Brenna Sabo, Sara Conroy, Garey Noritz, Madalynn Wendland, Linda P. Lowes

**Affiliations:** 1 Center for Gene Therapy, Nationwide Children’s Hospital, Columbus, Ohio, United States of America; 2 Department of Pediatrics, The Ohio State University, Columbus, Ohio, United States of America; 3 Center for Biostatistics, The Ohio State University Wexner Medical Center, Columbus, Ohio, United States of America; 4 Biostatistics Resource at Nationwide Children’s Hospital, Nationwide Children’s Hospital, Columbus, Ohio, United States of America; 5 Department of Pediatrics, Nationwide Children’s Hospital, Columbus, Ohio, United States of America; 6 School of Medicine, The Ohio State University, Columbus, Ohio, United States of America; 7 Doctor of Physical Therapy Program, Cleveland State University, Cleveland, Ohio, United States of America; IRCCS Medea: Istituto di Ricovero e Cura a Carattere Scientifico Eugenio Medea, ITALY

## Abstract

**Purpose:**

This single-arm prospective cohort study aimed to evaluate the feasibility and utility of in-home body weight support harness system (BWSS) use in children treated for spinal muscular atrophy (SMA).

**Methods:**

Individuals with 2 or 3 copies of *SMN2* who received pharmacotherapeutic treatment, had head control, and weight <50lbs were enrolled. Families were provided a BWSS and documented use. Motor outcome assessments were completed at baseline, month 3 and month 6. Families provided feedback in an end of study survey.

**Results:**

All 32 participants (2.9 (SD 1.9) yrs), improved or remained stable on all outcomes. Average reported frequency of use was 4.1(2.3) hrs/week. Controlling for other covariates, frequency of use explained over 70% of the variability in change scores. Family feedback was overwhelmingly positive.

**Conclusion:**

Use of in-home BWSS is a safe, feasible and useful option to increase exercise dosage after treatment in SMA and may help optimize motor abilities.

**Trial registration:**

Study registered with: Clinicaltrials.gov

Clinicaltrials.gov identifier: NCT05715749.

## Introduction

Spinal muscular atrophy (SMA) is a rare, autosomal recessive, neuromuscular disease involving the degeneration of lower motor neurons in the spinal cord resulting in progressive muscle atrophy [1–3]. SMA is most commonly caused by a homozygous mutation of exon 7 in the survival motor neuron 1 (*SMN1*) gene, disrupting production of survival motor neuron (SMN) protein; this protein is vital for the survival of motor neurons in the brain stem and spinal cord [4]. Disease severity is generally related to the number of *SMN2* genes, a “back-up” that produces a partially functional SMN protein [4]. Without treatment, individuals with SMA type 1 (2 copies *SMN2*) never achieve any major motor milestones, specifically never gain the ability to sit and typically require invasive ventilation or have died by 2 years of age [3]. Type 2 (3–4 copies *SMN2*) is a less severe phenotype in which the children gain the ability to sit but never walk without support [3, 4]. Since December 2016, the Food and Drug Administration (FDA) has approved three disease-modifying pharmacotherapeutic treatments (DMTs) for individuals with SMA: nusinersen (Biogen, Cambridge, USA), an intrathecal medication delivered every 4 months; onasemnogene abeparvovec-xioi (Novartis, Basel, Switzerland), a one-time intravenous gene replacement therapy; and risdiplam (Roche, Basel, Switzerland), a daily oral medication [5–7]. The success of these novel pharmacotherapeutics has changed the natural course of the disease by prolonging the lifespan and allowing the attainment of motor milestones never before achieved in individuals with SMA [8].

While evidence has shown that treatment with these DMTs can stabilize or even improve motor abilities, the magnitude of effect appears dependent, at least partially, on the age of treatment initiation and the extent of pretreatment symptoms [9, 10]. Motor unit loss may already be present at birth and will continue to progress until the treatment is received [8]. For most children living with SMA, treatments were approved and accessed well after the onset and progression of muscle weakness. Due to this early progressive weakness, individuals with SMA frequently have significant motor delay and are typically older and larger before they start attempting weight-bearing activities, if at all. Historically, physical therapy intervention for individuals with SMA was focused on management and prevention of secondary impairments (e.g. positioning, contracture management, etc.); however, with DMTs and the promise of motor neuron preservation, the intentional implementation of neuromotor habilitation, which may include the use of a partial body-weight support system (BWSS) has the potential to further optimize a child’s long-term functional gains [1, 10–12].

The use of BWSS in neuromotor rehabilitation is not novel [13–15]. Practice is important in the development of new skills, as demonstrated by typically developing toddlers taking over 2,300 steps each day while learning to walk, and these systems can allow a child with a motor delay to practice challenging skills in a partial weight-bearing environment [16]. However, traditional BWSS typically utilize a heavy, static device commonly positioned over a treadmill, allowing only uni-planar movement at prescribed speeds which has been shown to limit lasting functional carryover [14, 17]. They are also primarily only available in medical facilities, limiting the time children can spend safely practicing challenging activities. Aquatic therapy is another method of body weight supported exercise, but also requires access to specialized services and locations thus limiting its accessibility as well.

The purpose of our study was to evaluate the feasibility and utility of using an in-home portable multi-planar BWSS in children treated for SMA.

## Materials and methods

**Fig 1:**
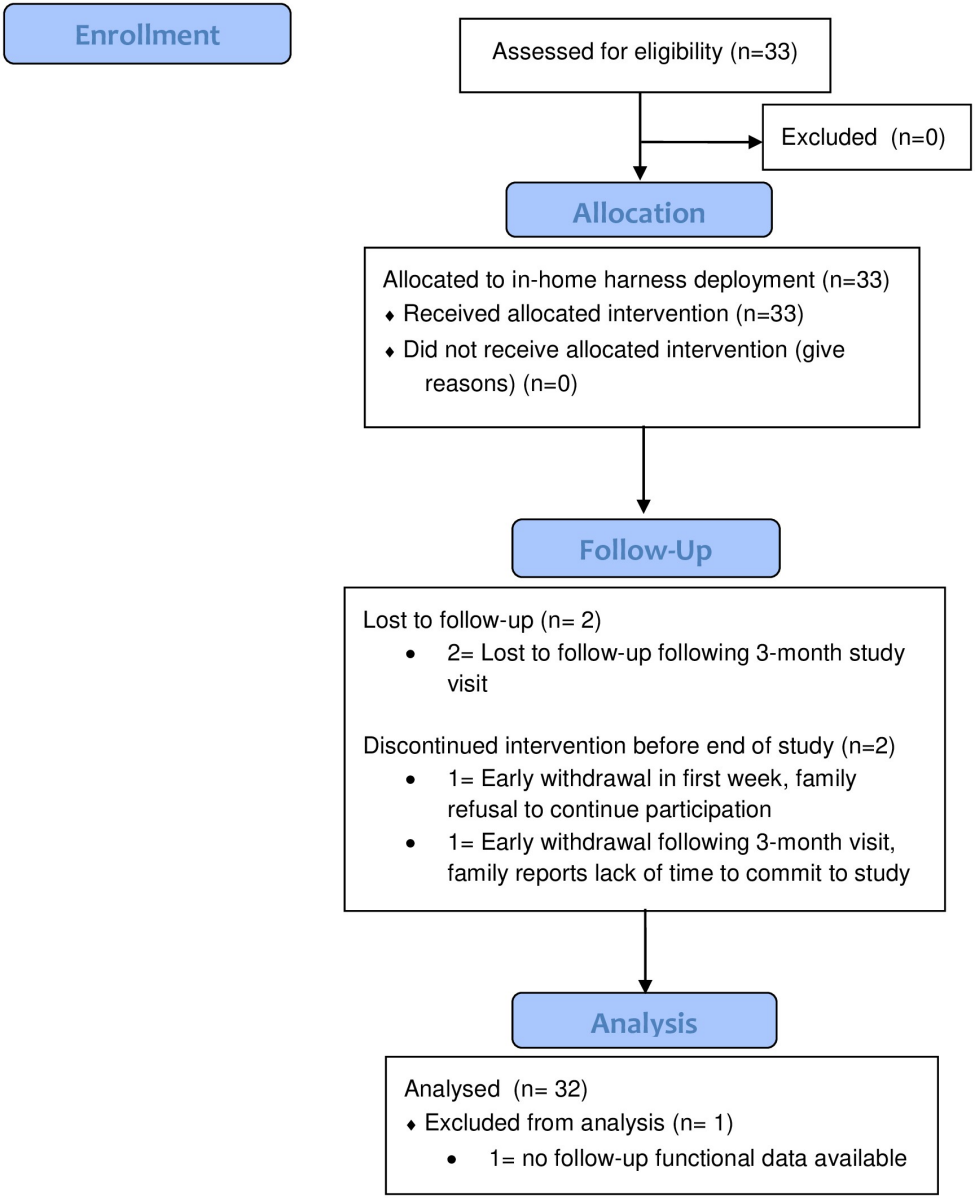
Participant recruitment and study flow diagram.

This study is designed as a prospective cohort study, following a one-group pretest-posttest design and was approved by the Institutional Review Board at The Abigail Wexner Research Institute at Nationwide Children’s Hospital (NCH). The authors confirm that this study for this intervention is registered; it was registered following initial enrollment as this is a small pilot study and recruitment was expected to be completed locally. Each participant’s legal guardian provided written informed consent, and written assent was obtained when appropriate; the individuals in this manuscript has given written informed consent (as outlined in PLOS consent form) to publish these case details. Given this was a pilot study and following CONSORT guidelines for feasibility and pilot studies, a formal sample size calculation was not conducted [18]. Participants were recruited as a sample of convenience through the NCH SMA clinic and word of mouth across expert SMA clinics in the United States between August 2018 and November 2019.Inclusion criteria were as follows: confirmed heterozygous mutation in *SMN1* gene and 2 or 3 copies of *SMN2* (historically, SMA Type 1 and Type 2), past or current treatment with nusinersen, onasemnogene abeparvovec, or risdiplam, upright head control (defined as the ability to lift head from full forward flexion), weight under or 50lbs, and confirmed motor delay. Participants were excluded if they had 4+ copies of *SMN2* (historically, SMA Type 3 and 4), there was evidence of lower limb injury or recent fracture, or in the opinion of the investigator, it was unsafe for the child to participate. CONSORT participant flow diagram provided in Fig 1.

### Design

**Fig 2:**
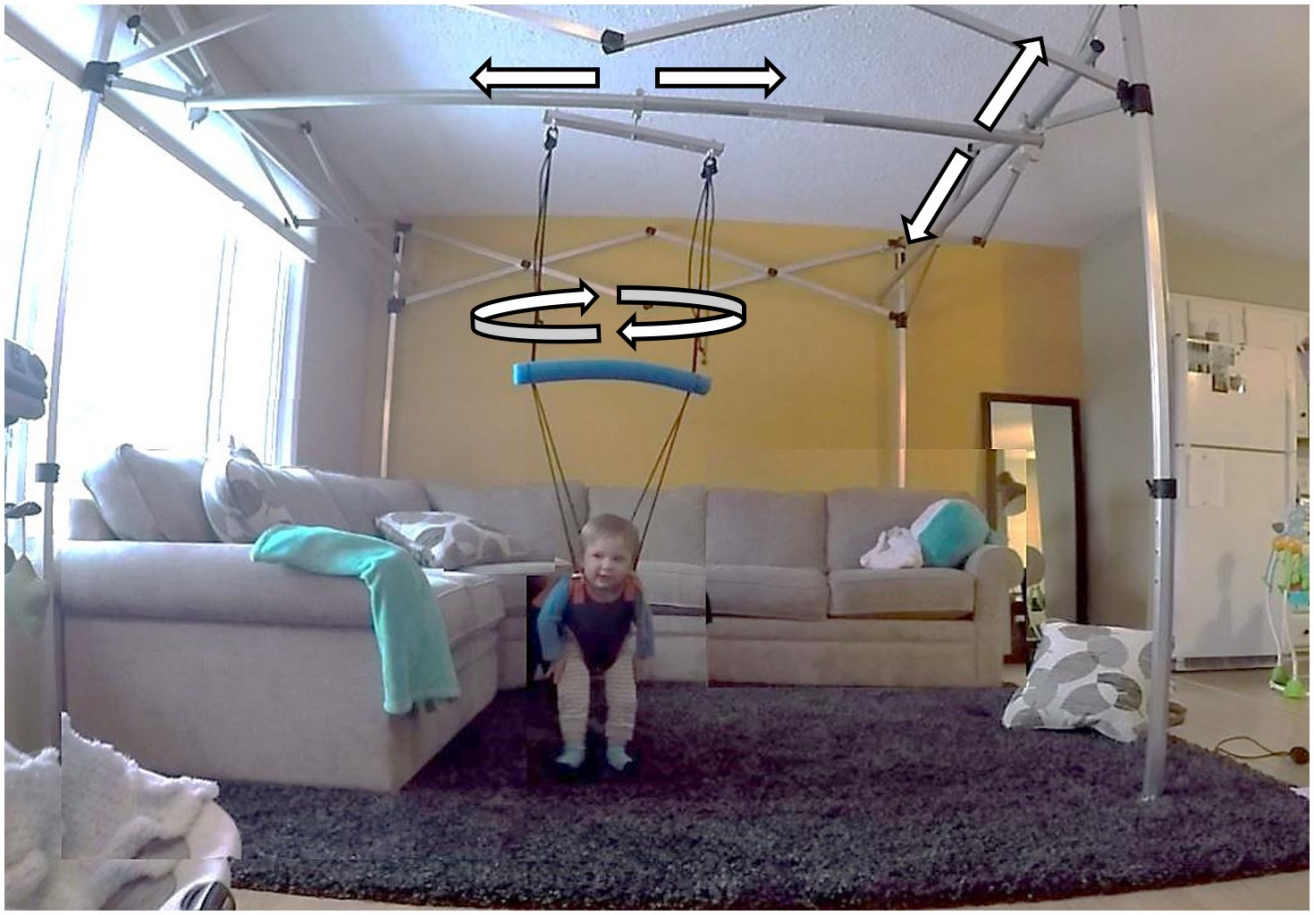
The PUMA portable body weight support harness system. The PUMA portable body weight support harness system (Enliten LLC, Delware, USA) expands to easily fit above and around household furniture.

**Fig 3:**
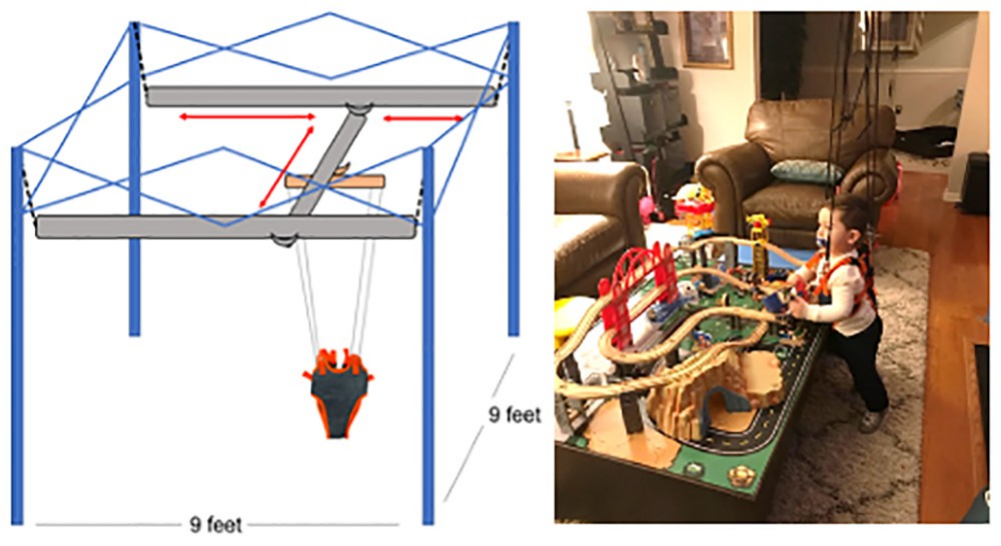
The PUMA integrates into the home environment. The PUMA allows children to access mobility within the full 9’x9’ footprint while in their home environment.

The Portable Mobility Aid for Children (PUMA) system (Enliten LLC, Newark, USA) used in this study is a commercially available, portable adjustable BWSS unit that occupies roughly a 9x9-foot footprint and can fit around and over household furniture (Figs 2 and 3). Resembling a collapsible camping tent frame, the BWSS includes inter-connecting cross bars that allows 360-degrees of mobility anywhere within the boundaries of the frame (Fig 3). The system provides a friction-limited glide, allowing children with little strength to propel themselves to move. Individuals are secured in a fabric harness and connected to the unit by attachment points on the front and back of the harness. The BWSS can safely hold an individual up to 60lbs and there are no restrictions based on height or age.

**Fig 4:**
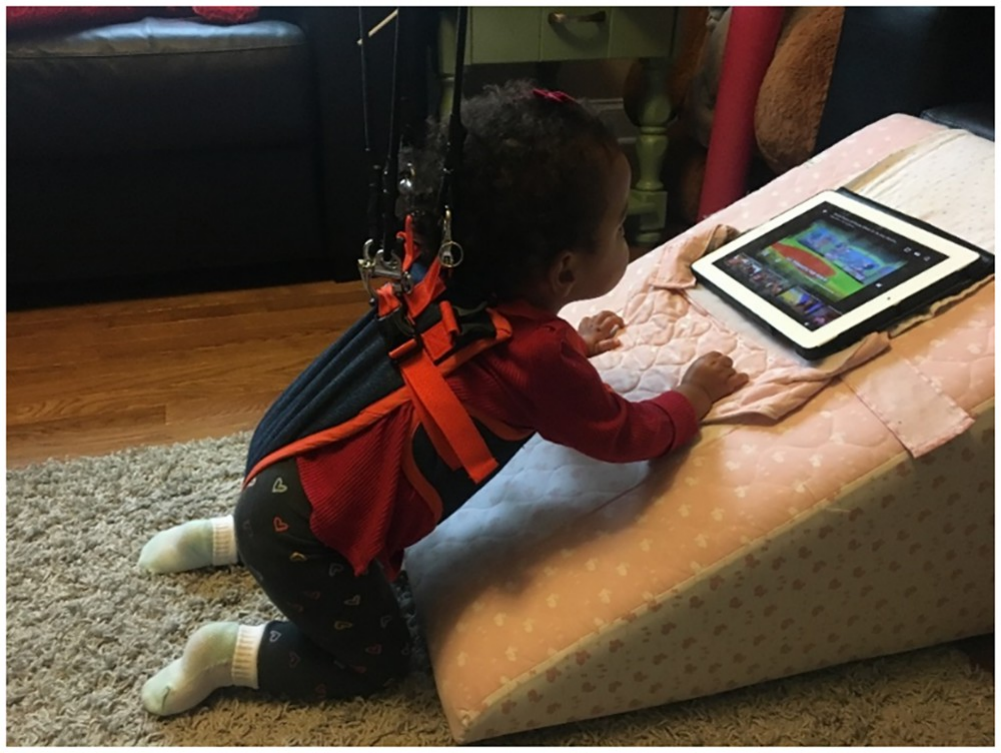
Support provided by the PUMA can be adjusted to consistently meet a child’s evolving needs. The counter-weight option allows more freedom for practicing challenging positions and transitions through all planes of movement.

This BWSS has two interchangeable support options: one that uses bungee cords and one that uses a counter-weight pulley system. The bungee option provides variable body weight support, providing more stability as the child is unable to completely collapse to the floor. The counter-weight option provides a set amount of desired support, which allows progression to the amount of support by changing the weight of the counterweight. It can be set to allow the child to reach the ground and thus practice transitions in and out of developmental positions (Fig 4). Families were provided with the bungee system at baseline and introduced to the counter-weight option at the mid-term visit; if and how each option was used was controlled by the family.

Families were encouraged to use the BWSS daily for 6 months. Investigators traveled to the family’s home to conduct baseline testing, set up the BWSS, and educate on proper use and safety considerations. Instruction manuals, safety inspection checklist, activity booklet with ideas for interactive play while using the BWSS, and activity logs to document frequency and duration of BWSS use were provided [19]. Families were permitted to continue any current habilitative therapies (e.g. physical therapy, aquatic therapy, etc.) or community activities/exercise and this information was documented.

Assessments of motor performance were completed by three physical therapist investigators at baseline, 3 months, and 6 months within a two-week window, with additional check-ins by family request. Activity logs documenting reported frequency of use were collected at month-3 and month-6 visits.

### Measures

Feasibility was quantified using a 5-question survey completed by families at the end of the study. Each family was asked to rate (1) the perceived impact of BWSS on motor and (2) non-motor skills, (3) the child’s perceived enjoyment using the system, (4) the ease of equipment use, and (5) if the family believed that use of the BWSS was beneficial on a 5-point Likert scale; scores of 4 or 5 were considered favorable, scores of 3 were considered neutral, and scores of 1 or 2 were considered unfavorable. The study was considered feasible if >80% of the items were scored 3, 4, or 5. This survey also included a section for comments. Study-related adverse events (AEs) and serious adverse events (SAEs) were collected through the duration of the study.

Utility of the BWSS system was measured by both reported frequency of use and change in motor function as defined by performance on The Neuromuscular Gross Motor Outcome (GRO), Hammersmith Functional Motor Scale Expanded (HFMSE), Revised Hammersmith Scale (RHS), the Gross Motor Subtest of the Bayley Scales of Infant and Toddler Development version 3.0 (Bayley GM), and World Health Organization Motor Milestone Checklist (WHO) [20–24]. All motor outcomes were administered using standardized testing procedures, as published by each study respectively. These motor outcomes are objective measures of motor function that, in general, rely on the administrator to assign a value of performance on each item (1 = able, 0 = unable, for Bayley and WHO; or 2 = able, 1 = able with compensations, 0 = unable, for GRO, HFMSE, and RHS). Participants were evaluated by the same investigator across all study visits and investigators were blinded to previous performance at subsequent visits. Since all participants were provided a BWSS, investigators were not blinded to the allocation of treatment but hourly documented use from activity logs was not calculated until the conclusion of the study.

The GRO is a 50-item performance-based outcome developed to quantify motor function across the span of age and abilities in individuals with neuromuscular disorders including SMA [20]. Items are organized in a developmental progression; many items can be scored spontaneously or are easily elicited. Ordinal level scoring allows participants to receive a 2 (able to complete the task), a 1 (able to partially complete the task or do so with compensation), or a 0 (unable to complete the task) for a total possible score of 100 points.

The original HFMSE and the RHS are disease-specific and performance-based outcomes originally developed to measure motor skill in untreated individuals with SMA [21, 22]. Item scoring is ordinal level with individuals achieving a 2 (able without compensations), 1 (able with compensations), or 0 (unable) for a total of 66 and 69 points for HFMSE and RHS, respectively. Historically, the HFMSE has been a standard motor outcome assessment for young children with SMA and the RHS is a revised version following Rasch analysis. These assessments require specific starting positions and an ability to follow discrete instructions. Due to the average age of individuals enrolled, administration was modified with all attempts to honor item-construct; cueing and scoring was kept consistent across all participants and visits. A similar approach to item administration has been reported in individuals with SMA under 2 years old [25].

The Bayley GM is a norm-referenced developmental assessment of gross motor skill in children 16 days– 3.5 years of age [23]. Items are ordered based on expected motor development and scored using a dichotomous scale of 0 (unable to demonstrate task) or 1 (able to demonstrate task). Raw scores are used to determine scaled scores, which compare the child’s performance to a normative sample of typically developing age-matched peers. All participants, regardless of age were provided a Bayley GM raw score due to delayed attainment of milestones.

The WHO is a brief checklist that surveys the attainment of major developmental gross motor milestones. The 6 milestones that make up the WHO range from sitting independently to walking independently. WHO was administered according to published guidelines [24].

### Data analysis

Age at enrollment is summarized using mean, standard deviation, and range. Age at DMT initiation, time since DMT initiation, baseline outcome scores, and change in outcome scores were summarized using median and inter-quartile range (IQR). Milestone achievement is presented as a count. Mann-Whitney U tests were used to compare groups by SMN2 copy number. Frequency of BWSS use is reported as mean, standard deviation (SD) and range, as is other weekly reported therapies. Pearson correlation coefficient was used to examine the relationship between other reported exercise and reported BWSS use, and the relationship between change in motor performance and reported BWSS use.

To evaluate the association of frequency of BWSS use and change in motor scores, four linear regression models for each outcome measure were used. The first model adjusted for baseline score. The second model included baseline score and reported additional therapies and exercises. The third model included age at baseline and time since treatment as potential confounders, as research suggests that children treated at a young age have the greatest potential for change and that a child makes significant gains early in the treatment period that may slow down or plateau over time [8, 9]. The final model added *SMN2* copy number to assess the impact on the estimated effect of frequency of use. Likelihood ratio tests were used to assess model improvement after adding variables. Analyses were conducted using SPSS (version 26) and R (version 1.2.5033).

## Results

Thirty-three participants were enrolled (15 female, 18 male) between September 2018 and December 2019, prior to widespread availability of newborn screening for SMA; 1 participant withdrew within one week of consent citing social considerations, 1 withdrew following the 3-month visit reporting a lack of time to commit to the study, and 2 additional participants were lost to follow-up following their 3-month visit. All subjects who received treatment with nusinersen had completed at least their four initial loading doses prior to enrollment and were established on a maintenance dosing schedule. Participant characteristics at baseline are presented in Table 1.

**Table 1:** Participant characteristics, motor performance at baseline and 6 months, and documented frequency of use.

**Baseline**	**Overall**	**2 copies *SMN2***	**3 copies *SMN2***	**P-value** ^d^ ^,^ ^e^
	**n = 33**	**n = 14**	**n = 19**	
Age (yrs)				
Mean (SD)	2.9 (1.9)	2.6 (1.3)	3.1 (2.2)	ns
Range	0.8, 9.4	0.8, 4.8	0.9, 9.4	
Age at Initiation of Treatment* (yrs)^a^	0.94 (0.40, 1.67)	0.38 (0.26, 0.48)	1.56 (0.96, 2.57)	<0.001
Time since treatment* (yrs)^a^	1.00 (0.49, 1.79)	1.35 (0.86, 3.49)	0.79 (0.40, 1.54)	ns
GRO^a^	40.0 (36.0,46.0)	38.0 (31.5, 40.8)	42.0 (36.0, 49.0)	ns
HFMSE^a^	20.0 (13.0, 27.0)	19.0 (8.5, 21.8)	20.0 (13.5, 31.0)	ns
RHS^a^	13.0 (10.0, 18.0)	13.5 (8.5, 16.0)	13.0 (10.5, 20.5)	ns
Bayley GM^a^				
Raw score	22.0 (21.0, 29.0)	22.0 (20.0, 22.8)	25.0 (21.0, 30.5)	ns
Scaled score	1 (1.0, 1.0)	1.0 (1.0, 1.0)	1.0 (1.0, 1.0)	ns
**6 months**	**Overall**	**2 copies**	**3 copies**	
	**n = 32**	**n = 14**	**n = 19**	
GRO score change from baseline^a^	4.5 (1.8, 8.0)	5.0 (3.0, 12.0)	4.0 (1.5, 6.5)	ns
HFMSE score change from baseline^a^	5.5 (2.0, 10.8)	8.0 (4.0, 10.0)	4.0 (1.5, 11.0)	ns
RHS score change from baseline^a^	3.5 (1.8, 7.5)	5.0 (2.0, 9.0)	3.0 (1.5, 7.0)	ns
Bayley GM change^a^				
Raw score	2.5 (1.0, 6.0)	4.0 (2.0, 6.0)	2.0 (1.0, 5.5)	ns
WHO change^b^				
No new milestones	22 (69%)	9 (28%)	13 (41%)	
1 new milestone	4 (13%)	1 (3%)	3 (9%)	
2 or more new milestones	6 (18%)	3 (9%)	3 (9%)	
Frequency of Use^c^	4.1 (2.3)	4.8 (2.3)	3.7 (2.2)	ns
	0.6, 9.5	1.8, 9.4	0.6, 9.5	

^a^Median (interquartile range),

^b^n(%),

^c^Mean (SD-standard deviation), range,

^d^Mann-Whitney U test,

^e^ns = not significant

*Treatment = treatment with DMT

GRO = Neuromuscular Gross Motor Outcome; HFMSE = Hammersmith Functional Motor Scale Expanded; RHS = Revised Hammersmith Scale; Bayley GM = Bayley Scales of Infant and Toddler Development, version 3, gross motor subtest

### Baseline characteristics

At enrollment, there was no significant difference in age or time since DMT initiation between copy number groups. The two youngest participants were under 1-year old at baseline and were 10 and 4-months post-DMT initiation. Average age at DMT initiation was 0.94 years (IQR, 0.40, 1.67); children with 2 copies of *SMN2* were treated at a younger age likely because the severity of this type leads to an earlier diagnosis. Twenty-four children (73%) were greater than 6 months from initial DMT and 16 children, or roughly half the cohort, were >1 year from initial DMT.

Baseline performance on GRO, HFMSE, RHS, and Bayley was similar by *SMN2* copy number (Table 1). All participants demonstrated a significant delay in gross motor development, receiving a Bayley GM scaled score of 1 (N = 32, 97%) or 4 (N = 1, 3%) at baseline. All 33 participants were able to sit independently, 6 participants were able to crawl, 4 participants stood with assistance, 1 was able to stand alone and 2 walked with assistance. None of the participants walked independently.

Reports of additional weekly therapies/exercise activities averaged 1.7 (SD 0.8) and ranged from 1–3.5 hours per week. Seven participants (22%) did not have access to skilled therapy services but reported at least 1 hour per week dedicated to exercise focused on promoting motor development.

### Longitudinal performance

Thirty-two of the 33 participants enrolled were included in longitudinal analysis; the participant that withdrew within one week of enrollment was excluded as there was no follow-up functional data available. For the 3 participants with missing 6-month visit data the 3-month assessment scores were imputed at 6-months to conservatively assume no additional changes were achieved. The average reported frequency of BWSS-use was 4.1 (SD 2.3) hours per week (range: 0.6–9.5) and was similar between *SMN2* copy number groups. There was no relationship between frequency of BWSS use and hours of additional therapies (S1a Fig).

Average change in performance on motor outcomes is presented in Table 1. There was no significant difference in the magnitude of change between *SMN2* copy number groups. Two participants (6%) improved Bayley GM scaled scores, increasing from 4 to 5 and 1 to 3; all other scaled scores remained stable at 1. Ten participants (31%) gained 19 new milestones: crawling (N = 5); standing with assistance (N = 6); and walking with assistance (N = 4) were the most frequently gained skills.

**Fig 5:**
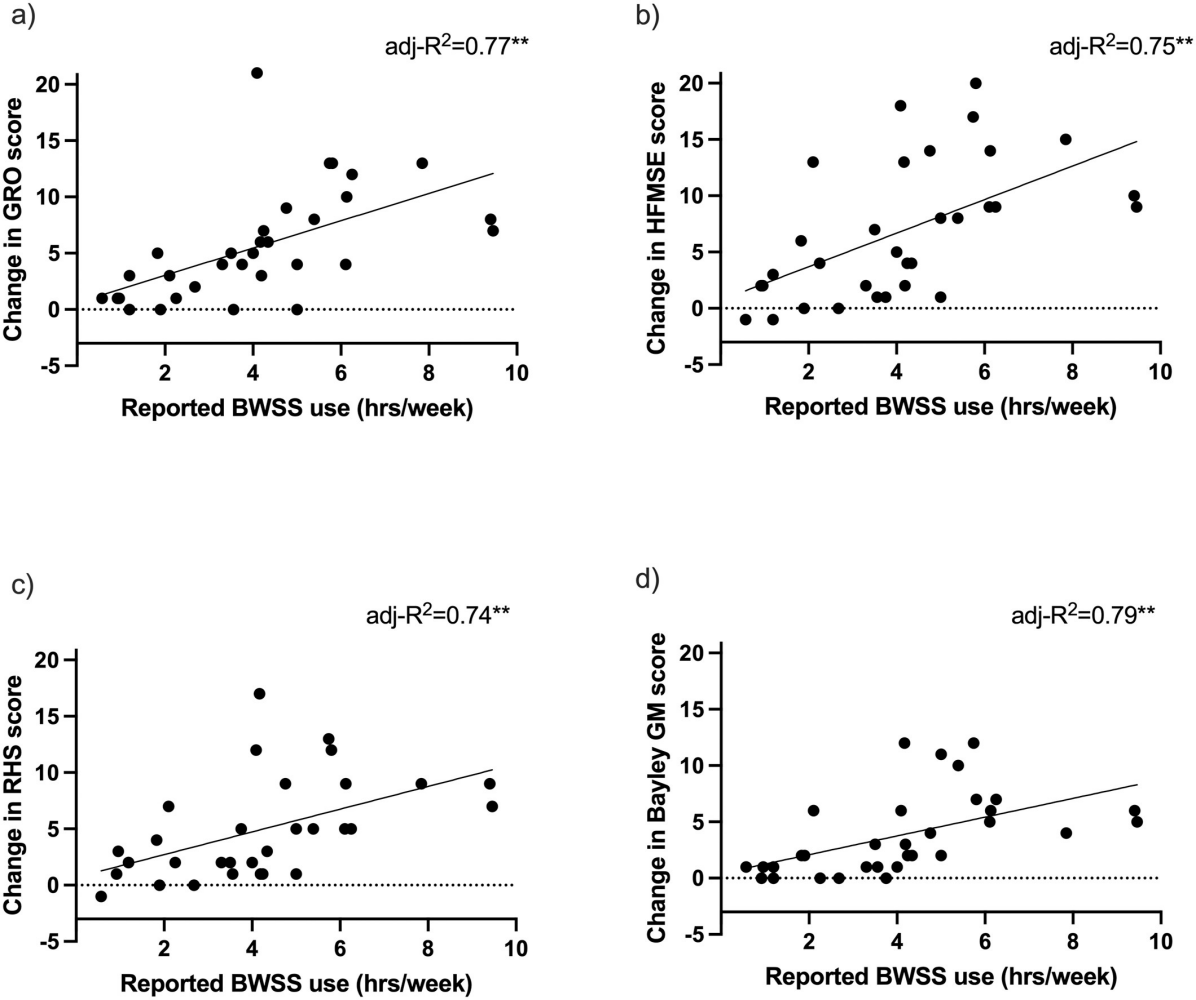
Frequency of BWSS use by 6-month change in motor performance. Scatter plots with lines of best fit,depicting reported frequency of BWSS use by 6-month change in performance on the GRO (a), HFMSE (b), RHS (c), and Bayley GM (d). After controlling for baseline score, 74–79% of the quantity of change in each assessment can by explained by reported frequency of BWSS use (adjusted R2).

Individual change on each outcome by reported frequency of BWSS-use (average hours/week) are presented in Fig 5. There were significant correlations between frequency of BWSS use and change in performance on motor outcomes (Pearson’s r of 0.67, 0.67, 0.62, and 0.63 for GRO, HFMSE, RHS, and Bayley GM-raw respectively; all p<0.01). There was no significant relationship between change in motor performance and additional exercise/therapies (S1b–S1e Fig). Using linear regression modeling, the average frequency of BWSS use explained between 74 to 79% of the change from baseline after controlling for the baseline score (Table 2, Fig 5). The model also suggests that for each additional hour of harness use per week there was an expected mean change of 0.81 (95% CI 0.64, 0.97) points on the GRO, 0.85 (95% CI 0.67, 1.03) on the HFMSE, 1.02 (95% CI 0.80, 1.25) on the RHS, and 1.07 raw points (95% CI 0.86, 1.28) on the Bayley GM-raw after controlling for baseline scores. Adding additional therapy time, age, time since treatment and SMN2 copy number did not significantly improve the model fit or explain more variability in the change in outcome scores (Table 2).

**Table 2:** Estimated mean and 95% CI for change in outcome measure score comparing participants with an additional hour of harness use.

Outcome	Model 1^a^^,^^e^	Adjusted R-squared	Model 2^b^^,^^e^	Adjusted R-squared	Model 3^c^^,^^e^	Adjusted R-squared	Model 4^d^^,^^e^	Adjusted R-squared
**GRO**	0.81 (0.64,0.97)	0.77	0.84 (0.67, 1.01)	0.78	0.84 (0.65,1.04)	0.77	0.85 (0.64,1.06)	0.76
**HFMSE**	0.85 (0.67,1.03)	0.75	0.88 (0.69, 1.06)	0.76	0.95 (0.73,1.17)	0.76	0.94 (0.71,1.17)	0.75
**RHS**	1.02 (0.8,1.25)	0.74	1.06 (0.84, 1.29)	0.74	1.17 (0.91,1.43)	0.76	1.16 (0.89,1.42)	0.75
**Bayley GM raw**	1.07 (0.86,1.28)	0.79	1.06 (0.85, 1.29)	0.78	1.13 (0.9,1.37)	0.78	1.14 (0.89,1.40)	0.77

Adjusted R-squared, proportion of variability in change in outcome measures explained by variables in linear regression models

CI: Confidence interval

^a^ Model adjusted for baseline outcome measure only

^b^ Model adjusted for baseline outcome measure and additional hours in therapy

^c^ Model adjusted for baseline outcome measure, additional hours in therapy, age and time since treatment at follow up

^d^ Model adjusted for baseline outcome measure, additional hours in therapy, age, time since treatment at follow up, and SMN2 copy number

^e^ All model estimates had p<0.00

GRO = Neuromuscular Gross Motor Outcome; HFMSE = Hammersmith Functional Motor Scales Expanded; RHS = Revised Hammersmith Scale; Bayley GM = Bayley Scales of Infant and Toddler Development, version 3, gross motor subtes*t*

### Study completion survey

**Fig 6:**
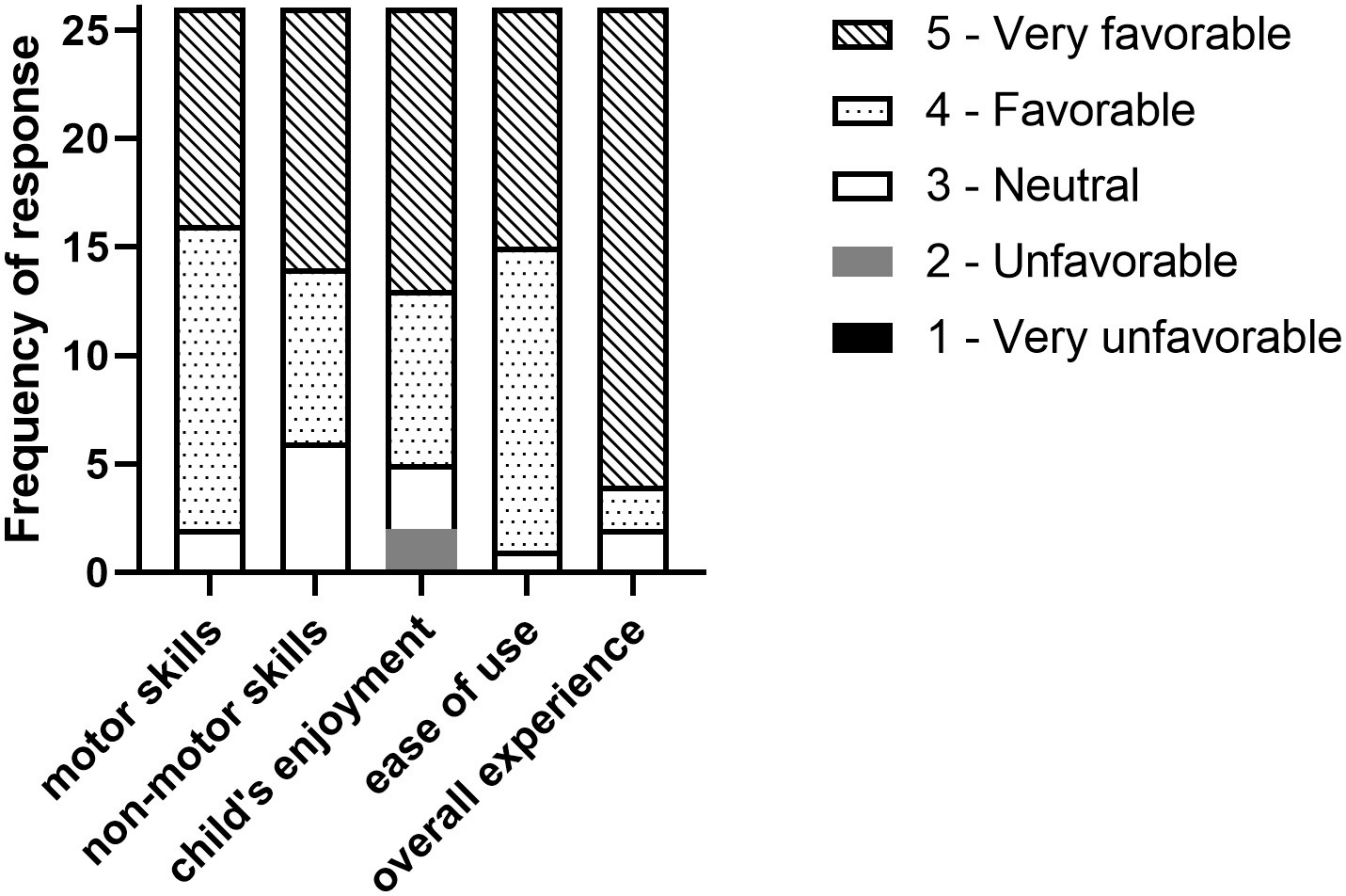
Study completion survey results. The study completion survey was completed by 26 of the 32 families that finished the study. On average, families reported a favorable response with average Likert scores of 4.3(0.6), 4.2(0.8), 4.2(0.9), 4.4(0.6), and 4.8(0.6) for questions 1–5, respectively. Unfavorable responses were related to discomfort caused by the fabric harness (3/32 families).

Twenty-six of the 32 families returned the study completion survey. Overall, families reported a highly favorable response, with 114/130 (88%) of responses coded with a 4 or 5. Further, 14 (10%) of responses were neutral and 2 (2%) responses were unfavorable. Three families with boys reported discomfort from forces through the child’s groin that resolved once removed from the harness, accounting for 2 of the unfavorable and 1 of the neutral responses. Despite these instances of discomfort, there were no study-related AEs or SAEs. Detailed scoring is presented in Fig 6 and additional family comments can be found in S1 File.

## Discussion

The BWSS is a safe, feasible, and useful method to increase exercise dosage and may help optimize gross motor abilities in this cohort of young children with SMA, post DMT-treatment. Disease modifying therapies (DMTs) have changed the traditional phenotype of individuals with SMA, most dramatically when implemented early. Treatment response can be more modest and/or plateau when implemented later in infancy or childhood [8]. Physical therapy interventions must also adapt to this changing landscape to optimize the impact on patients’ lives and outcomes. Previously, physical therapy intervention for individuals with more severe forms of SMA (2 and 3 copies *SMN2*) focused on management and prevention of secondary impairments with the expectation that motor function would decline with disease progression. As the first DMT was approved for use in 2016, there is limited data yet on the impact of exercise or physical therapy to optimize treatment effect and motor outcomes.

Our cohort of young children who have been treated for SMA (2 or 3 copies *SMN2*; historically, Type 1 and 2), demonstrated stable or improved motor function including achievement of new motor milestones over the 6-month study period. This is not unexpected, as children with SMA treated with DMTs at very young ages are expected to gain strength and function from those treatments, particularly immediately following treatment. However, as our cohort is not limited to children who recently initiated DMTs (16 children [48%] were ≥1-year post-treatment) or initiated DMTs within the first weeks of life (average age at treatment initiation is 0.94 years), our findings suggest that regular BWSS use may help optimize treatment gains in children even 2+ years post-treatment. After adjusting for confounding factors that may influence change in performance over time (e.g., frequency of use, age, time since treatment initiation, and *SMN2* copy number), frequency of BWSS use was the only significant contributor to measured functional improvements in our sample. While this study was not designed to evaluate efficacy of BWSS use and strong causal relationships between BWSS frequency of use and motor improvement cannot be determined without a control group, we did find a linear relationship between frequency of BWSS use and positive change in motor performance. Thus, this data suggests that children with similar baseline characteristics to our sample may also experience benefit from frequent BWSS use post-treatment as well.

This study supports the use of an in-home BWSS as a safe and feasible modality for young children who have been treated for SMA. There were no study-related AEs for any child. Families felt confident and safe using the BWSS across the study with the initial in-home deployment, safety check, and treatment activity materials provided. In our cohort, feasibility of in-home BWSS was supported by 88% positive feedback on use by families, surpassing the 80% benchmark, and 97% of families actively using the system across the 6-month study for an average of 4 hours per week. Specific feedback from families was related to the improved access to a therapeutic modality within the home and ease of use and implementation. While in this study we quantified feasibility based on feedback provided by families, we recognize that other real-world factors including price and availability are considerations for widespread implementation across family homes and healthcare facilities.

Traditional outpatient therapy models can be limited in frequency and duration for many reasons (e.g., family schedules, transportation, availability of services). Thus, it can be difficult to receive an adequate treatment dosage to make meaningful gains in strength and endurance, as exercising once a week is likely insufficient [26]. Our study supports this assertion, as additional therapies or exercise dosage alone were not significant contributors to the measured changes in function. In this cohort, the upper range of total exercise time nearly tripled with the inclusion of in-home BWSS use providing convenient access and an option to increase exercise dosage [15]. In addition, while this study was not designed or powered to determine optimal treatment dosage, our findings may help support benefit to intensive therapy dosage.

In conjunction with motor skills gained, parental survey results suggest that in-home BWSS may provide additional non-motor benefits. Improved access to toys and the home environment, optimized play with peers and siblings, and increased inclusion in family activities, were recurring themes of overwhelmingly positive parent report. It is also possible that the opportunity for early self-initiated mobility and self-driven exploration of the environment provided by the BWSS could facilitate the development of cognitive, social, language, and other skills that have been shown to be experience dependent, as variability of experience is especially important during critical periods of development [27, 28].

There are limitations to this study. Firstly, this study was completed with a small sample size and was only 6 months in duration. We also relied on parent report to document the number of hours spent using the system, which could affect the accuracy of the data. Similarly, the high rate of positive response reported on the end of study survey may be the result of parental reporting bias, as only 26 of the 32 families returned the survey. Additionally, other unknown determinants (e.g. genetic, social) may have influenced performance over time that were not accounted for in the regression analysis. To note, the 3 families that reported less satisfaction with “ease of use” and “enjoyment” all had male children and were the oldest (ages 6, 6, and 9) and largest in the study. The families reported that groin discomfort limited the length of time spent in the fabric harness. These three male participants also had the greatest lower extremity contractures when compared to the remainder of the cohort. Larger and older individuals with similar characteristics may benefit from an alternative style of harness that would limit discomfort, as they may enjoy the social interaction of being upright and having an independent means of mobility, albeit in a confined space.

The limitations and experience gleaned from this study provide considerations for future research and recommendations for families considering this modality option. In this cohort, most families continued to use the bungee system even after the counterweight system was introduced. The counterweight system can be more difficult to operate independently, requires more constant effort from the child, and was reported as less fun. Those that reported success with the counter-weight system predominantly used it with an in-home therapist. A future trial that includes additional in-person therapist-assisted sessions may determine if using the counterweight system in place of the bungee system could further improve motor function. Further, a study of longer duration and larger enrollment may lead to more consistent results. Additionally, while static standing frames are commonly recommended for individuals with SMA that are unable to stand or walk without support, a retrospective study suggests motor performance is not associated with standing frame use [29]. Dynamic weight bearing opportunities, as with BWSS use, may include the additional benefit of motor improvement as individuals are actively exercising muscles while in a supported upright position and may also impact bone strength and development, as the muscle tension strains from mechanical loading during dynamic weight bearing are suggested to provide additional benefits towards bone health and bone development [30, 31].

## Conclusion

A BWSS system should be considered as a therapeutic modality for young children treated for SMA who meet the inclusion criteria of head control and do not exceed the weight limit recommended by the manufacturer, as this modality has shown to be safe, feasible, well-tolerate by families, and may be useful for optimizing gross motor abilities.

## Supporting information

S1 Fig Relationship between change in motor performance and variables other than frequency of use. There was no relationship between a) reported frequency of BWSS and other reported hours of exercise/therapies, or other reported hours of exercise/therapies and change in performance on the b) GRO, c) HFMSE, d) RHS, e) Bayley. Correlations were calculated using Pearson correlation coefficient, r, with reported p-values.
(TIF)

S1 File Additional comments on experience using BWSS provided by families following the 6-month study period. (PDF)

S1 Protocol Use of body-weight support harness systems in children treated for spinal muscular atrophy. (DOCX)

S1 Checklist CONSORT 2010 checklist of information to include when reporting a randomised trial*. (DOC)
