# Supplementary figures and images for: Feasibility and utility of in-home body weight support harness system use in young children treated for spinal muscular atrophy: A single-arm prospective cohort study
Source: PLoS One. 2024 Mar 19;19(3):e0300244. doi: 10.1371/journal.pone.0300244 (PMC10950233; doi:10.1371/journal.pone.0300244)

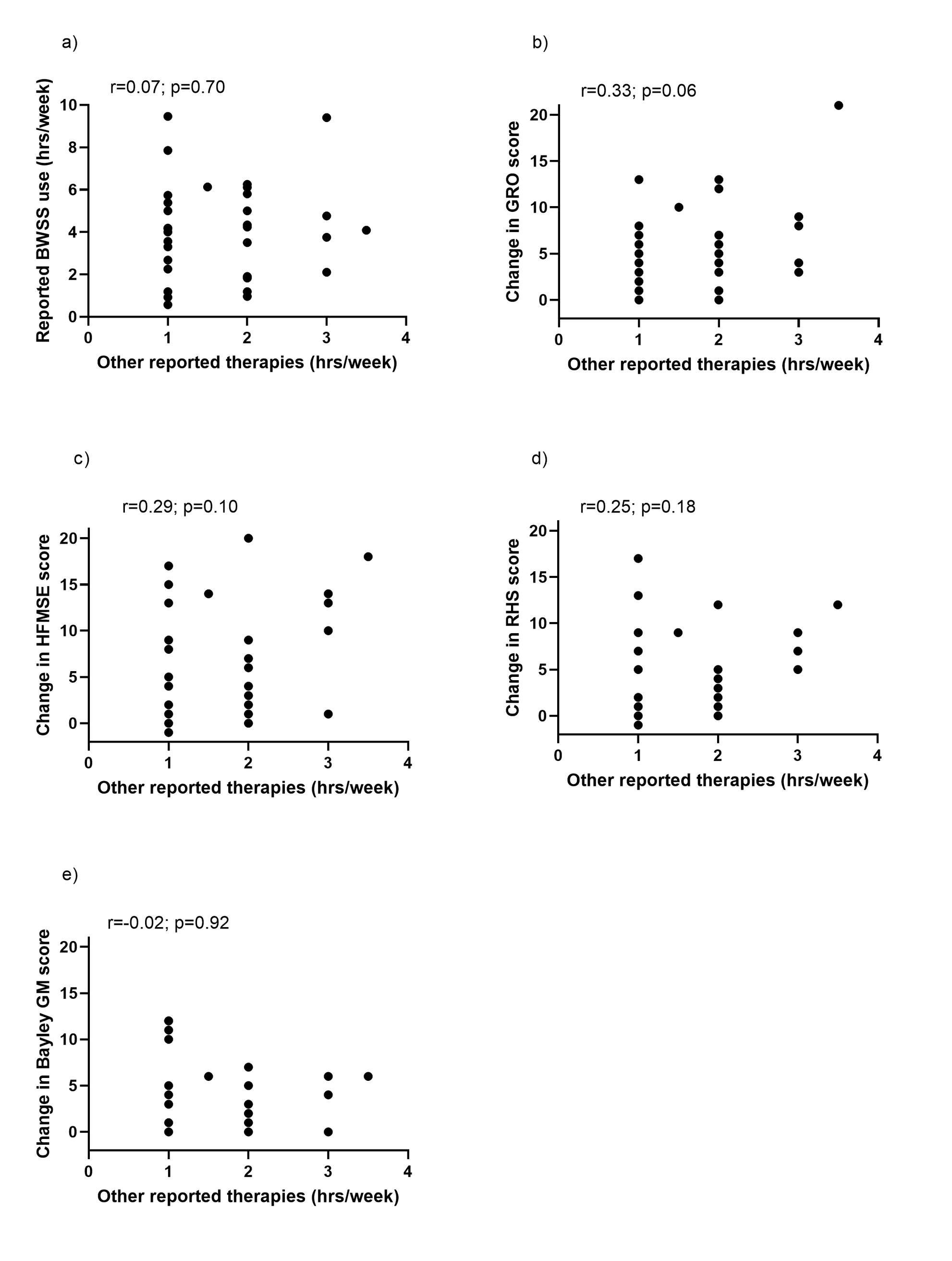

Supplement: S1 Fig — There was no relationship between a) reported frequency of BWSS and other reported hours of exercise/therapies, or other reported hours of exercise/therapies and change in performance on the b) GRO, c) HFMSE, d) RHS, e) Bayley. Correlations were calculated using Pearson correlation coefficient, r, with reported p-values. (TIF) [file pone.0300244.s001.tif]
