# Supplementary material for: Feasibility and utility of in-home body weight support harness system use in young children treated for spinal muscular atrophy: A single-arm prospective cohort study
Source: PLoS One. 2024 Mar 19;19(3):e0300244. doi: 10.1371/journal.pone.0300244 (PMC10950233; doi:10.1371/journal.pone.0300244)
Supplement: S1 File — (PDF) [file pone.0300244.s002.pdf]

“My daughter loves the harness! It allowed her to be a ballerina with her siblings. It also strengthened her legs tremendously. We especially appreciated the harness in the colder months when we aren’t outside. I would highly recommend the harness to anyone in need.”

“It took a week or so before [my son] started enjoying time in the harness, at first with bungee and then again when we switched to counterweight, but after that period he enjoyed the freedom and independence it gave him to play and to move around with his sister. Almost daily he asked “go in jumpy?” (what he called the harness). We truly believe it played an important roll in the improvement of his motor skills – with the combo of medicine, therapies, in just 6 months he gained the ability to kneel, push up to sitting, crawl, pull up to standing, stand almost on his own, great with braces and even take a few steps with braces!”

“[My daughter] was able to experience play in a vertical position for a long period of time like any other child. Her happiness, freedom were the greatest joy that a mom can have.”

“The harness system has been amazing and great help for our daughter, she doesn’t care for the suit up of the harness, but overall she enjoys the abilities she gains while in the harness system.”

“[My son] asks to be in his harness at least 2x a day. He gets very excited to be able to play and interact – that wouldn’t be possible without this system. We have noticed a major change in his leg strength with lifting them. As well as now when he is out of his harness he is more apt to want to take steps and “walk”. I would strongly recommend this system for any family working on strengthening muscles. The social interaction is also crucial, as [he] is now able to participate and play ring-around, join in on dance parties, and mimic everyone around him.”

“[My son] really enjoyed being in harness, but could only tolerate around 30-40 min at a time.”

“[My son] was not able to use the harness much, his body was just too awkward so he could not tolerate the discomfort caused by the harness”
