# Supplementary material for: Feasibility and utility of in-home body weight support harness system use in young children treated for spinal muscular atrophy: A single-arm prospective cohort study
Source: PLoS One. 2024 Mar 19;19(3):e0300244. doi: 10.1371/journal.pone.0300244 (PMC10950233; doi:10.1371/journal.pone.0300244)
Supplement: S1 Protocol — (DOCX) [file pone.0300244.s003.docx]

**PROTOCOL TITLE:**

Use of body-weight support harness systems in children treated for spinal muscular atrophy

**PRINCIPAL INVESTIGATOR:**

Name: Megan Iammarino PT, DPT

Department/Center: Center for Gene Therapy

Telephone Number: 614-722-6813

Email Address: megan.iammarino@nationwidechildrens.org

**VERSION NUMBER/DATE:**

Version 2.2/ 04Aug2020

**REVISION HISTORY**

| **Revision #** | **Version Date** | **Summary of Changes** | **Consent Change?** |
| --- | --- | --- | --- |
| 2.1 | 04Aug2020 | Transferred from old IRB format to new format using Form 503 | No |
| 2.2 | 04Aug2020 | Updated to include option for subjects to submit video evidence of motor skill attainment for remote visits | Yes |
|  |  |  |  |
|  |  |  |  |
|  |  |  |  |

# Study Summary

| **Study Title** | Exploring the feasibility and utility of in-home body weight support harness system use in children treated for spinal muscular atrophy |
| --- | --- |
| **Study Design** | Pilot interventional study |
| **Primary Objective** | Investigate whether BWSS is a feasible option for in-home exercise and investigate the use of home-based functional exercise using a body weight support harness system (BWSS) on motor performance in children treated for spinal muscular atrophy |
| **Secondary Objective(s)** | Explore influence of frequency of use on change in motor performance over 6 months. |
| **Research Intervention(s)/ Investigational Agent(s)** | Enliten, LLC portable body weight support harness system (BWSS) |
| **IND/IDE #** |  |
| **Study Population** | Children who have been treated for spinal muscular atrophy |
| **Sample Size** | Up to 50 children |
| **Study Duration for individual participants** | On-going; each child will be followed for six months with possibility of extension. |
| **Study Specific Abbreviations/ Definitions** | BWSS – body weight support harness system  SMA – spinal muscular atrophy |

# Objectives

- 1. The purpose of the study is to investigate the feasibility and utility of using an in-home body weight support harness system (BWSS) to promote motor performance in children who have been treated for spinal muscular atrophy. Our primary aim is to explore whether BWSS is a feasible modality for in-home use and whether it impacts motor performance. Our secondary aims are to determine whether frequency of BWSS impacts treatment effect.

# Background

- 1. Spinal muscular atrophy (SMA) is an autosomal recessive disease involving the degeneration of lower motor neurons in the spinal cord that results in progressive muscle atrophy.1 SMA is characterized into four main phenotypes based on age of onset and highest motor milestone achieved: type I (unable to sit unassisted); type II (able to sit unassisted but unable to stand or walk independently); type III (walks independently); and type IV (adult-onset).2 Historically, a multidisciplinary treatment approach is aimed at limiting the trajectory of decline through proactive management of clinical manifestations.3,4 With newer technologies providing improved supportive care, it has been hypothesized that the expected phenotype for all types of SMA is evolving toward improved survival and optimized function.5 This maintenance approach to symptomatic management is also being driven to change as recent therapeutic advancements are making their way through the clinical trial pipeline. In December 2016, Spinraza® (nusinersin) became the first US FDA approved drug available for the treatment of SMA.6 This was quickly followed by the promising preliminary results for AVXS-101 gene replacement therapy in children with SMA type I, which reported subject achievement of gross motor milestones beyond what would be expected for their diagnosis.7 Both therapies target the preservation of existing motor neuron connections in the spinal cord and provide greater evidence for a changing phenotype of symptom stabilization and functional improvement that will require a new approach to clinical care.
  2. Physical therapists play a key role in the evaluation and management of musculoskeletal manifestations and functional impairments in children and adults with SMA. Recently published care guidelines stratify suggested categories of physical therapy assessment and interventions by functional ability (non-sitters, sitters, and ambulatory).3 These guidelines were created prior to this new treatment-era of SMA and are aimed at supporting the highest level of function, not towards facilitating development of strength, skill, and more advanced motor milestones. The reported success of SMA drug therapies suggest physical therapy interventions may now be aimed at motor milestone and gross motor development.
  3. Typically developing children naturally build strength and motor skills by exploring their environment through curiosity-driven mobility. Deficits in muscle strength limit independent and self-guided mobility in children with SMA due to the fact that their muscles are unable to support the whole weight of their own body. By unweighting a portion of their total body weight, children with SMA will be provided with an opportunity to begin activating the muscles in their legs and trunk, something they would otherwise be unable to do. Daily in-home use of a body weight support harness system may assist these children in building strength and developing new skills.

# Study Endpoints

- 1. Primary endpoints for feasibility include using a 5-question Likert-style scale survey of patient reported experience and documented frequency and duration of BWSS use. Primary endpoints for utility are assessments of gross motor function and include The World Health Organization’s gross motor developmental milestones, Bayley Scales of Infant Development v.3 Gross Motor subtests, Hammersmith Functional Motor Scale Expanded and/or Revised, and Neuromuscular Gross Motor Outcome.

# Study Intervention/Investigational Agent

- 1. Name of interventional agent: The Portable Mobility Aid for Children (PUMA); Body weight support harness system manufactured by Enliten, LLC
  2. The PUMA is a commercially available device that can safely be used to promote development in any child with a developmental delay. It provides mechanical support of a child’s body while allowing vertical and horizontal movement within the system’s frame. The BWSS was designed by an engineering company (Enliten, LLC). The BWSS is a metal structure that is connected to a harness worn by a child. The structure consists of four 7ft metal support beams (“legs”) that create a functional square footprint of 9ft by 9ft and adjustable heights up to 7ft 2in (Appendix D). The four legs are connected at the top by interconnected beams such that the system looks like a four legged square tent with flat top and no cover. A child wears a secure cloth harness that is connected to a moveable beam connected to the top of the metal structure. The connection between child/harness and metal structure is via springs, which provide body-weight support up to 50lbs. The moveable beam allows for movement throughout the square footprint. The secure cloth harness cradles the child’s entire torso for stability and comfort and will be adjusted according to each body type to provide safe movement.

Information regarding the concept, safety, and design of the BWSS as provided by Enliten LLC, can be found in Appendix E. Drug/Device Handling: If the research involves drugs or device, describe your plans to store, handle, and administer those drugs or devices so that they will be used only on subjects and be used only by authorized investigators.

# Procedures Involved*

- 1. This is a voluntary study investigating the feasibility and effects of a home-based functional exercise program using a body weight support harness system (BWSS) on motor performance in children treated for spinal muscular atrophy. Subjects will be provided with one portable BWSS, manufactured by Enliten LLC, to be used in their home for a period of 6 months. Subjects will engage in a BWSS use and document frequency / duration of use.
  2. **Screening/Baseline**: Subject demographic information and equipment safety check will be completed prior to the initial in-home visit. Baseline assessment of motor function may be completed at any time within a 3 week window prior to or during the initial in-home visit.

**Initial Visit (in-home):** A study team member will travel to the subject’s home to complete the BWSS set up. The BWSS Inspection Check List will be reviewed and signed by the responsible participating family member and the study team member. Pictures or video of the final set-up will be taken and stored in the subjects file. The participating family members will be trained on how to securely strap their child into the harness and on appropriate activities to engage in with their child while in the harness. They will be instructed on frequency and duration of treatment time and asked to document this information in the Daily Activity Log. They may be asked to attach an activity monitor to the harness.

Prior to completion of this visit, the following documentation will be completed: 1) Informed Consent; 2) BWSS Inspection Check List; 3) Pictures or video of BWSS set-up in home.

A study team member will contact the family between the baseline and midpoint assessment visit to ensure the family is comfortable with BWSS use. If the family has questions or concerns, they will be addressed by the study team member via Skype or video conference phone call. If determined necessary by the PI, a study team member will travel to the family’s home for an additional in-home visit.

**Midpoint Assessment Visit**: A study team member will complete functional outcome assessments and evaluate compliance with usage schedule. Daily Activity Log will be checked and completion will be documented.

Families will be given the option to complete the midpoint functional assessments during an in-home visit or during a regularly scheduled clinic or research appointment at NCH. Midpoint assessment visits may also occur remotely using approved video conferencing services. Photos and videos of the child performing motor skills will be accepted to incorporate into the midpoint assessment up to one week after the date of visit. Photo and video submissions will only be accepted for assessments performed remotely.

**Final Visit**: A study team member will complete final functional outcome assessments. Families will be given the option to complete the final functional assessments during the in-home visit or during a regularly scheduled clinic or research appointment at NCH. Final assessment visits may also occur remotely using approved video conferencing services. Photos and videos of the child performing motor skills will be accepted to incorporate into the final assessment up to one week after the date of visit. Photo and video submissions will only be accepted for assessments performed remotely.

Families will be provided with an opportunity to enter the Optional Enrollment Extension phase (see 6.2.1 point below).

If the family would like to continue enrollment but the BWSS is already assigned to another subject on the waitlist, the Final Visit will be completed as detailed in 9.2.1 and the family will be placed on the waitlist for re-enrollment.

6.2.1 **Final Visit - Optional Enrollment Extension:** Should the family choose to continue enrollment following the 6-month finial visit, the BWSS will remain in the family’s home until the family withdraws from the study or until BWSS equipment is needed for use with another subject.

Families will be asked to continue documenting BWSS use in Daily Activity Logs. Functional assessments may be performed, either in the home or during a regularly scheduled clinic or research appointment at every 6-9 months.

6.2.2 **Final Visit without Enrollment Extension:** A study team member will return to the family’s home to dismantle the BWSS unit. Prior to disassembly, a final equipment check using the BWSS safety Inspection Check List will be completed, reviewed, and signed by the study team member and responsible parent. Daily activity log will be checked and completion will be documented

- 1. All functional outcome assessments will be video recorded. Parents may also submit video evidence of motor skill attainment within 7 days of any remote study visit, should the child not perform on the date of the assessment due to child’s mood or behavior.
     - Parents will submit video evidence through secured email communication. Submitted videos will be stored on secured NCH research drive, alongside other assessment videos.
  2. FFamilies will be educated on appropriate activities to help engage with their child in the harness.

Each family will receive an Activity Ideas booklet to reference throughout the duration of the study (Appendix F).

- 1. Families will be asked to fill out the Daily Activity Log (Appendix G) each time the harness is used. The log will include information on the duration and frequency of BWSS use, activities completed with child while in BWSS, and any observed changes in child’s motor function
  2. Families may be asked to attach a motion sensor to the harness system.
  3. **Liability Waiver and Legal Agreement**: Families are to review and sign a liability waiver prior to in-home equipment set-up. This documentation to be created by Nationwide Children’s Hospital Corporate Legal Services department and addresses the expectations and responsibilities of the families to maintain the integrity of the BWSS for the duration of the study
  4. **Safety Considerations**
     - **Equipment Inspection**
       - Equipment will be required to pass study team member inspection using the BWSS Inspection Checklist provided by Enliten, LLC. (Appendix A)
       - Equipment safety checks will occur at screening (prior to in-home set up), baseline, and final visit. Photographs or video of in-home set up will be included in safety documentation.
       - It will be required that the BWSS Inspection Check List be completed by the study team member and reviewed and signed-off by the study team member and parent at baseline and final study visit.
     - **Adult Supervision**
       - Families are required to supervise their child while using the BWSS at all times.
     - **Family Training**
       - Families will be trained on use of the BWSS on their first home visit. Training will be conducted by an investigator on the study team and will involve: an overview of BWSS use; instructions on safely securing the child into the harness; application and technique for use of the bungee system; application and technique for use of counter-weight system; and adjusting the amount of body weight support.
       - Each family will receive an instruction manual detailing how to safely adjust the harness and amount of body weight support provided by the harness, to reference for the duration of the study (provided by Enliten, LLC; Appendix B and Appendix C).
       - Families will be instructed to leave the system as it was set-up in their home throughout the duration of the study. If the family needs to move or take down the BWSS, a safety check by a study team member will need to be completed prior to resuming use. This can be done as either an in-home visit or via a video conference call.
       - If needed, a member of the study team may call the subject’s parents to troubleshoot any technical difficulties that may arise. If the difficulties are not resolved over the phone, a member of the study team may schedule an in-home visit with the family. The family will be instructed not to use the system if they have any concerns about safety.

# Data and Specimen Banking*

- 1. N/A – no specimens will be taken or stored in this study
  2. All original copies of collected data including demographics, consent and liability waivers, functional outcome assessments, and completed Daily Activity Logs, will be stored in a secured and locked cabinet on hospital property. Electronic data and recorded videos and stored on encrypted password-secured hard drives that will be kept in a locked cabinet on hospital property. Access to study documentation and data will be limited to investigators listed on the IRB.

# Sharing of Results with Subjects*

- 1. Pre- and post-study assessment results may be shared with family upon request, at the completion of the study. They will be shared with the family verbally or through secured email correspondence.

# Study Timelines*

- 1. Data will be collected for a period of up to 2 years, with subjects enrolled in the interventional period for a duration of 6 months. Data collection of functional outcomes will occur at baseline, a midpoint assessment, and final visit at month six, +2 weeks for each timepoint.
  2. We anticipate all subjects be enrolled within one year of study start date and anticipate completion of study including primary analysis to be complete within two years of the study start date.

# Inclusion and Exclusion Criteria*

- 1. Subjects will be screened during regular clinic visits, by review of inclusion/exclusion criteria in correspondence with family, and/or prior to enrollment and informed consent process.
  2. **Inclusion criteria:** Able to safely fit in pediatric harness; genetic diagnosis of spinal muscular atrophy, delay in standing or walking, able to right head from full flexion, treatment with a disease modifying agent

**Exclusion criteria:** At any point during interventional period of the study, a body weight greater than 50 pounds (lbs) or 22.68 kilograms (kg); evidence of current or previous unresolved lower limb injury, current or recurrent fractures within the past 6 monhts that could affect performance or study procedures; medical condition or developmental history (i.e. concomitant illness, behavioral disorder, prematurity, uncontrolled seizure disorder) that in the opinion of the investigator, make it unsafe for the subject to participate or could impair study results

- 1. We will include individuals who are not yet adults (infants and children).

# Vulnerable Populations*

- 1. The research subjects will be minors. HRP-416 has been consulted. Consenting procedures are as follows:
     - The child’s parent or guardian will provide written informed consent according to institutional guidelines. If the child is over 7 years of age, they will provide written assent for participation. The study procedures, risks, and benefits to participation will be explained to the parent by a member of study staff. The parent will have the opportunity to ask questions and have their questions answered. If the subject and parent agree to participate upon full understanding of the study requirements, they will provide written informed consent.

# Local Number of Subjects

- 1. We expect 15 subjects to be enrolled locally.

# Recruitment Methods

- 1. Subjects will be recruited through Nationwide Children’s Hospital Spinal Muscular Atrophy clinic, through IRB approved flyers, and by word of mouth.

# Withdrawal of Subjects*

- 1. Participation in this study is optional for the family and subject; families are given the option to withdrawal at any time. Withdrawal from this study will not affect any care provided to the patient at Nationwide Children’s Hospital. Subjects may choose to withdrawal due to lack of time/scheduling conflicts, unrelated injury of subject, outside treatments that may affect BWSS use (i.e. surgery, serical casting, etc.)
  2. Final determination of family and subject eligibility is at the discretion of the PI. If at any point during the study, the patient is determined to be un-safe while in the BWSS (e.g. change in body type or health status, un-safe practices of family noted during in-home visits) the PI may withdrawal the family from the study
  3. If subjects are withdrawn from the study, they will be instructed to discontinue use of BWSS and a clinical evaluator will come to their home to disassemble harness system and complete final safety checks.

# Risks to Subjects*

- 1. Risks to subjects is no greater than risks associated with general activity in children with SMA. These include the child becoming tired or fussy, bone fracture, sore muscles, and tired head or neck which may result in airway occlusion. Families are required to provide constant adult supervision to mitigate this last risk.
  2. Risks associated with equipment use include: the child falling if not placed in the harness correctly, entanglement due to BWSS misuse or equipment malfunction. When used properly, there are no reports of BWSS causing harm to a child or adult when used as indicated.

# Potential Benefits to Subjects*

- 1. Subjects may experience a new way of moving and interacting with family members and peers. Use of BWSS may increase frequency of motor skill practice and facilitate motor skill development.

# Data Management* and Confidentiality

- 1. Baseline and Final visit assessment scores will be compared. We will explore factors that may impact outcomes including reported frequency of use. Subjects may be stratified into groups based on such factors and sample group change averages will be compared.
  2. All source form data will be stored in locked cabinets in a locked office for the duration of the study. Electronic data entry will be stored on secured NCH research drive (RESLowes) and will be password protected.
  3. Data will be stored indefinitely
  4. Access to data: only investigators listed on IRB will be provided access to study materials.

# Provisions to Monitor the Data to Ensure the Safety of Subjects*

- 1. Should injury occur as a result of BWSS use, PI will be notified immediately and will review the cause and severity of the injury. If necessary, all other subjects will be notified of cause/effect of injury and if determined to be the safest outcome, study will be terminated and all equipment will be returned to site.

# Provisions to Protect the Privacy Interests of Subjects

- 1. Privacy: Subjects will be asked to complete NCH Neurology Media Consent form which allows them to determine how their image will be used or disseminated. Families may opt out of videotaped assessments through this form. Additionally, The study staff will be visiting the subjects’ homes, allowing them to receive highly personalized, innovative services in a completely private environment with no need to travel (travel is challenging for these families). Every effort will be made to protect the privacy of these subjects and their families.
  2. Making subject feel at ease: The families in this study are facing a very challenging diagnosis. By offering these patients a treatment that can strengthen their child and slow progress of the disease, this study will potentially be improving their lives. It is our experience that these families are very much at ease with the therapy teams that they work with.

# Compensation for Research-Related Injury

- 1. There will be no compensation to the family for research-related injury. Please see section 6.5 Liability Waiver

# Economic Burden to Subjects

- 1. Subjects will not be responsible for any costs to participate in this study

# Consent Process

- 1. The child’s parent or guardian will provide written informed consent according to institutional guidelines. If the child is over 7 years of age, they will provide written assent for participation. The study procedures, risks, and benefits to participation will be explained to the parent by a member of study staff. The parent will have the opportunity to ask questions and have their questions answered. If the subject and parent agree to participate upon full understanding of the study requirements, they will provide written informed consent.
  2. Consenting will occur at first study visit in the family’s home or during screening process during regularly scheduled clinic visits.

# Process to Document Consent in Writing

- 1. We will be following HRP-091 Documentation of Consent.

# Setting

- 1. Research visits will occur in the families’ homes or at Nationwide Children’s Hospital during regularly scheduled clinic visits.

# Resources Available

- 1. We do not anticipate any difficulty reaching full enrollment. NCH SMA clinics occur twice monthly with roughly 8 patients per clinic (patients seen on a 6-month or 1-year basis).

# Multi-Site Research*

- 1. *N/A – this is a single site study*

# ****27.0 Protected Health Information Recording****

1. **Indicate which subject identifiers will be recorded for this research.**

Name

Complete Address

Telephone or Fax Number

Social Security Number (do not check if only used for ClinCard)

Dates (treatment dates, birth date, date of death)

Email address , IP address or url

Medical Record Number or other account number

Health Plan Beneficiary Identification Number

Full face photographic images and/or any comparable images (x-rays)

Account Numbers

Certificate/License Numbers

Vehicle Identifiers and Serial Numbers (e.g. VINs, License Plate Numbers)

Device Identifiers and Serial Numbers

Biometric identifiers, including finger and voice prints

Other number, characteristic or code that could be used to identify an individual

None (Complete De-identification Certification Form)

**2.0  Check the appropriate category and attach the required form* on the Local Site Documents, #3. Other Documents, page of the application.  (Choose one.)**

Patient Authorization will be obtained. (Include the appropriate HIPAA language (see Section 14 of consent template) in the consent form OR attach the HRP-900, HIPAA AUTHORIZATION form.)

Protocol meets the criteria for waiver of authorization. (Attach the HRP-901, WAIVER OF HIPAA AUTHORIZATION REQUEST form.)

Protocol is using de-identified information. (Attach the HRP-902, DE-IDENTIFICATION CERTIFICATION form.) (Checked "None" in 1.0 above)

Protocol involves research on decedents. (Attach the HRP-903, RESEARCH ON DECEDENTS REQUEST form.)

Protocol is using a limited data set and data use agreement. (Contact the Office of Technology Commercialization to initiate a Limited Data Use Agreement.

***Find the HIPAA forms in the IRB Website Library, Templates.**

**Attach the appropriate HIPAA form on the “Local Site Documents, #3. Other Documents”, page of the application.**

1. **How long will identifying information on each participant be maintained?**

**Identifying information will be stored indefinitely**

1. **Describe any plans to code identifiable information collected about each participant.**

Subjects will be given a study ID which will be documented in a password secured enrollment log. All study case report forms will be linked to the subject using their individual study ID number, not their name or MRN.

1. **Check each box that describes steps that will be taken to safeguard the confidentiality of information collected for this research:**

X **Research records will be stored in a locked cabinet in a secure location**

X **Research records will be stored in a password-protected computer file**

X **The list linking the assigned code number to the individual subject will be maintained separately from the other research data**

X **Only certified research personnel will be given access to identifiable subject information**

**6.0 Describe the provisions included in the protocol to protect the privacy interests of subjects, where "privacy interests" refer to the interest of individuals in being left alone, limiting access to them, and limiting access to their information. (This is not the same provision to maintain the confidentiality of data.)**

Subjects are entitled to withdrawal from the study at any point in time.

**Confidential Health Information**

1. **Please mark all categories that reflect the nature of health information to be accessed and used as part of this research.**

Demographics (age, gender, educational level)

Diagnosis

Laboratory reports

Radiology reports

Discharge summaries

Procedures/Treatments received

Dates related to course of treatment (admission, surgery, discharge)

Billing information

Names of drugs and/or devices used as part of treatment

Location of treatment

Name of treatment provider

Surgical reports

Other information related to course of treatment

None

1. Please discuss why it is necessary to access and review the health information noted in your response above.

Timing since initiation of treatment may be a factor in subject response to intervention; this will be explored during data analysis.

3.0 Is the health information to be accessed and reviewed the minimal necessary to achieve the goals of this research?  Yes  No

4.0 Will it be necessary to record information of a sensitive nature?  Yes  No

5.0 Do you plan to obtain a federally-issued Certificate of Confidentiality as a means of protecting the confidentiality of the information collected?  Yes  No
